# Multi‐Omics Analyses on Maya‐Land Tomatoes Shed Light on Plant Strategies to Thrive in High Temperatures

**DOI:** 10.1111/ppl.70429

**Published:** 2025-07-27

**Authors:** Luis F. Maceda‐López, Annie Espinal‐Centeno, Jose Juan Ordaz‐Ortiz, Alfredo Cruz‐Ramírez

**Affiliations:** ^1^ Metabolomics and Mass Spectrometry Laboratory, Unidad de Genómica Avanzada, Centro de Investigación y Estudios Avanzados Instituto Politécnico Nacional Irapuato Guanajuato Mexico; ^2^ Molecular and Developmental Complexity Group, Unidad de Genómica Avanzada, Centro de Investigación y Estudios Avanzados Instituto Politécnico Nacional Irapuato Guanajuato Mexico

**Keywords:** heat stress, metabolomics, native varieties, tomato, transcriptomics

## Abstract

Heat stress limits tomato yield and quality. Deciphering the key genes and metabolites related to heat tolerance is essential for selecting tolerant varieties. In this study, we profiled the transcriptomes and metabolomes of roots and shoots in response to heat stress from a Maya‐land ecotype of heirloom tomato (Calkiní), which grows in lowlands and elevated temperatures. We compared them with those of another heirloom ecotype that grows in highlands and lower temperatures (Acaxochitlán). In our omics approach, several transcripts encode enzymes that participate in diverse biosynthetic pathways and produce differentially accumulated metabolites. Although both ecotypes display programs to deal with heat stress, the roots of the Calkiní ecotype showed an increased accumulation of diverse metabolites and the up‐regulation of key genes involved in molecular and physiological strategies to cope with heat stress. One of the key findings of this study is the elevated accumulation of transcripts and metabolites associated with phenylpropanoid and suberin biosynthesis in the heat‐resistant ecotype. Such increases correlate with substantial suberin deposition in the exodermis and endodermis of Calkiní roots, with enhanced gibberellin accumulation in the meristematic zone under heat stress. Additionally, we found differential expression and accumulation of metabolites and genes involved in the PA‐GAPC‐NFY heat stress tolerance pathway among both ecotypes. Our work strengthens the importance of studying Mexican native tomato varieties and ecotypes to identify traits that allow plants to cope with diverse biotic and abiotic stresses, specifically providing insights into the genetic and metabolic pathways linked to heat tolerance among ecotypes and tissues.

## 1 Introduction

Plants regularly deal with abiotic stresses, such as drought, heat, cold, salinity, and flooding, which determine their growth and development. These abiotic factors limit crop yields and affect quality, productivity, and global food production (Driedonks et al. 2018; Zhang et al. 2022). Heat stress is the major limiting factor affecting crop production among these abiotic stresses. With the rise of temperatures caused by global warming, the urge to maintain plant growth and development under high temperatures becomes challenging for several crops and most parts of the world (Alsamir et al. 2021; Bineau et al. 2021). This is also the case for tomato ( *Solanum lycopersicum* ), one of the most cultivated horticultural crops that reached 186.1 million tons in 2022 in a cultivation area of 4.91 million hectares, based on data from the Food and Agricultural Organization of the United Nations. Despite its adaptability to various environmental conditions, increasing temperatures are predicted to cause a projected decrease in tomato production of 6% by 2050 (Cammarano et al. 2022). Additionally, temperatures are expected to increase between 1.5°C and 11°C by the end of the 21st century (Stainforth et al. 2005; Bita and Gerats 2013). Tomato plants are susceptible to heat stress during different vegetative and reproductive growth stages, significantly affecting pollen viability, seed development, root elongation, fruit setting, and quality (Alsamir et al. 2021). Also, several recent studies have focused on tomato plants' transcriptomic and metabolic responses to heat stress and have shed light on the molecular mechanisms developed by tomato cultivars to deal with heat stress. For instance, metabolites like GABA, myo‐inositol, sucrose, and various phenolic compounds increase immediately after heat stress or during leaf recovery, suggesting a dynamic metabolomic response (Paupière et al. 2020). Elevated temperatures also negatively impact tomato nutritional quality by reducing bioactive compounds, such as carotenoids and vitamin C, especially during advanced stages of fruit development. Additionally, heat stress alters membrane lipid composition, essential for maintaining thermotolerance, notably by decreasing lipid unsaturation (Almeida et al. 2021).

Furthermore, another study on tomato fruits from heat‐stress‐sensitive and ‐tolerant plants found that bioactive metabolite accumulation, including carotenoids and polyphenolics, supports stress tolerance and enhances the fruit's nutritional and therapeutic value. This highlights the value of untargeted metabolomics for identifying biomarker metabolites for breeding programs focused on developing more resilient tomato varieties (Singh et al. 2024).

Some studies have also investigated responses to combined stresses, identifying key regulatory mechanisms and genes. For example, a study on the combined effects of salt and heat stress found that salt stress primarily affects the roots, causing osmotic and ionic stress (Sousa et al. 2022). In contrast, heat stress impacts above‐ground parts, leading to more severe physiological changes (Li et al. 2024). The transcriptomic and metabolomic responses under combined stress closely resembled those observed under heat stress alone, suggesting that heat stress is the dominant factor when tomato plants face both salt and heat stress simultaneously (Li et al. 2024). However, many of these studies have been conducted on hybrid and commercial tomato plants. Mexico is the center of tomato domestication and has hundreds of native tomato varieties that have evolved to adapt and thrive in diverse and contrasting climates and soil compositions (Lobato Ortiz et al. 2012; Razifard et al. 2020); we considered that such a rich genetic diversity is an extraordinary resource for studying varieties and ecotypes adapted to low and high temperatures. We took advantage of such diversity to collect two ecotypes of tomato from the Heirloom variety, one of them tolerant to heat stress that grows in the lowlands of Calkiní in the state of Campeche (former Maya land) and the other one that grows in the highlands and lower temperatures in Acaxochitlán in the state of Hidalgo.

Our study compares the transcriptional and metabolic programs of roots and shoots of plants from the two ecotypes in response to heat stress. Here, we describe that hundreds of genes are differentially expressed as well as dozens of differentially accumulated metabolites among tissues and ecotypes in response to heat stress. An integrated approach of the transcriptomic and metabolomic profiles allowed us to identify that the roots of the Calkiní ecotype (from now on CAL) produce and accumulate more suberin and gibberellins than those of the Acaxochitlán ecotype (from now on ACA) in response to heat stress.

## 2 Materials and Methods

### 2.1 Biological Material

We selected two ecotypes of tomato heirloom that display contrasting responses to heat stress. One of the ecotypes, known as the Rosapa'ak tomato, was collected from Calkiní, Yucatan, Mexico, where the average temperature during the cultivation season is 34°C; this is a heat‐tolerant ecotype (CONAGUA 2024). A heat‐sensitive ecotype was collected from Acaxochitlán Hidalgo, Mexico, during the cultivation season, with an average temperature of 15°C (CONAGUA 2024). The seeds were surface sterilized with 70% (v/v) ethanol for 2 min, followed by 3% (v/v) sodium hypochlorite for 10 min and washed with sterilized distilled water at least four times. To induce germination, seeds were placed in a dark room for 48 h at 4°C and 96 h at 25°C. Germinated seedlings were placed on MS (Murashige and Skoog 1962) medium (pH 5.7) containing 3% sucrose and 0.8% agar in a growth chamber under a 16‐h light/8‐h dark cycle at 23°C. Then, control plants were maintained at 23°C while another group of plants from both ecotypes was grown for 3 h at 37°C to induce heat stress and returned to 23°C every day for 12 days for RNA extraction and 14 days for metabolite extraction.

### 2.2 RNA Isolation and Sequencing

Leaves and roots were collected from control plants and heat‐stressed plants of both ecotypes. The harvested samples were frozen in liquid nitrogen and pulverized, and total RNA was isolated from 100 mg of homogenized tissue. The total RNA isolation was performed using a PureLink Plant RNA Reagent (Invitrogen) following the protocol indicated in the Kit. The quality of RNA was assessed by agarose gel electrophoresis and concentration and purity parameters (A260/280 and A260/230) using a NanoDrop One^C^ UV–Vis Spectrophotometer (Thermo‐Scientific). The total RNA was used as input material for cDNA library preparation using the MGIEasy RNA total Library kit (MGI Tech) and was sequenced using the MGISeq 2000 platform (MGI Tech) to obtain 150‐bp paired‐end reads.

### 2.3 De Novo Transcriptome Assembly

Raw data from all these short‐read cDNA libraries were used for a de novo transcriptome assembly and functional annotation. Before assembly, a quality control of the sequenced reads was carried out. Raw reads were filtered for high‐quality sequences using the quality Control.py.script from Next‐Generation Sequencing (NGS) toolkits (https://github.com/Czh3/NGSTools/blob/master/qualityControl.py, accessed on September 27, 2022) with the default settings. Overlapping reads were merged into a single longer read using the Seqprep program (https://github.com/jstjohn/SeqPrep, accessed on October 25, 2022). The resulting reads were mapped to the genome assembly for *S. lycopersicum* version SL4.0 using Burrows‐Wheeler Aligner (BWA; Li and Durbin 2010).

### 2.4 Expression Analysis

We calculated the expected counts, TPM and FPKM to quantify gene expression levels. A gene was considered as expressed if, in each biological replicate, the gene had a normalized expected count value of 10 or higher. Differentially expressed genes were detected using the R package “DESeq2” (Love et al. 2014), with the following thresholds: *padj* < 0.05; |log2FC| > 2.

### 2.5 Metabolite Extraction and UHPLC–MS Analysis

One hundred mg of leaf and root samples for each ecotype and treatment were taken and ground to a powder. The powdered samples were dissolved in 4 mL 80% (v/v) methanol for metabolite extraction, vortexed for 1 min, and sonicated on ice at 50 Hz for 20 min. After that, the samples were centrifuged at 2000 *g* for 10 min; the supernatant was transferred to a new tube and stored in an ultra‐low temperature refrigerator at −80°C. All samples were vacuum dried (miVac, Genevac) at 30°C for 30 min and kept at −80°C until further analysis. All samples were resuspended in 1 mL of acetonitrile/ultra‐pure water 50:50 (v/v) and filtered through a membrane of 0.2 μm (PTFE, Agilent Technologies). Five quality control (QC) samples were prepared to account for instrument drift and system calibration during analysis in UHPLC‐QTOF‐HRMS; each QC sample was prepared by mixing homogeneously all sample extracts into a new single vial. QC samples were distributed at the injection run list's beginning, middle, and end. Extraction blanks were also considered during the experiment. For UHPLC–MS analysis, all samples (including QC and blank extraction) were injected according to a randomized list order on a UPLC (Acquity I class, Waters) coupled with an orthogonal QTOF mass spectrometer (SYNAPT G1 HDMS, Waters).

Chromatographic separation was achieved on a reversed‐phase BEH C18 column (2.1 mm × 150 mm, 1.7 μm, Waters) maintained at 40°C during chromatographic separation. Auto‐sampling of 10 μL per sample was injected. Compounds were eluted using ultra‐pure water with 0.1% (v/v) formic acid (solvent A) and acetonitrile with 0.1% (v/v) formic acid (solvent B) with a flow rate of 0.3 mL min^−1^ with the following gradient program: From 0 to 1 min, 15% B; 1 to 25 min, 15% B; 25 to 27 min, 100% B; 27 to 27.1 min, 100% B; 27.1 to 30 min, 15% B. The mass spectrometer mass range was set from 50 to 1500 Da. Both ionization modes were injected separately. For negative electrospray ionization (ESI) mode, the conditions were set as follows: Capillary voltage 2 kV; cone voltage 40 V; source temperature 150°C; cone gas flow 20 L h^−1^; desolvation temperature 350°C; desolvation gas flow 600 L h^−1^. For positive ESI mode: Capillary voltage 3 kV; cone voltage 40 V; source temperature 130°C; desolvation temperature 350°C; desolvation gas flow 700 L h^−1^. Leucine‐Enkephalin (2 ng mL^−1^) was infused as LockSpray reference internal mass calibrant at a flow rate of 5 μL min^−1^, and its signal was monitored every 10 s. The data format was collected in a continuum mode with an MS scan time of 1.5 s. In both the positive and negative ionization modes, data were acquired in MS^E^ experiments using Argon as the collision gas with collision energy in the trap region of 6 eV (Function 1, low energy) and ranging from 20 to 40 eV (Function 2, high voltage).

### 2.6 Data Analysis

Positive and negative electrospray ionization mode raw data were imported to Progenesis QI for small molecules software (Non‐Linear Dynamics, Waters) for automatic alignment, normalization, deconvolution, and compound pre‐identification over all samples. The RT range for the pre‐identification method was limited from 1 to 25 min. Pre‐identification was performed using ChemSpider (http://www.chemspider.com/), Plant Metabolic Network (https://www.plantcyc.org/), KEGG (http://www.genome.jp/kegg/), HMDB (http://www.hmdb.ca/), and ChEBI (https://www.ebi.ac.uk/chebi/) with a minimum match of 90% for precursor ions. MS/MS data and isotope distribution were included for increasing match score values. Statistics and graphics were performed using MetaboAnalyst 6.0 (Pang et al. 2024). Differential metabolites were identified via one‐way ANOVA, PCA, PLS‐DA, and OPLS‐DA analyses.

### 2.7 Combined Transcriptome and Metabolome Analysis

The combined transcriptomics and metabolomics analysis was conducted with PaintOmics (https://paintomics.uv.es/; Liu et al. 2022). Transcriptomics and metabolomics datasets with *S. lycopersicum* gene IDs and metabolite names were uploaded into PaintOmics by choosing *S. lycopersicum* as an organism and KEGG, Reactome, and MapMan as databases.

### 2.8 Immunofluorescence Assay for Gibberellin Detection

Primary root tips of 14 days post germination roots from CAL and ACA ecotypes, from control plants or heat‐stressed plants, were collected to investigate endogenous GA gradients. The roots were fixed overnight at 4°C in 4% paraformaldehyde (PFA) in phosphate‐buffered saline (PBS). After fixation, they were washed three times with PBS and incubated in ClearSee solution (10% xylitol, 15% sodium deoxycholate, 25% urea) for 3 days with daily solution changes. Following clearing, the roots were rewashed and placed on slides to dry for 2 h at room temperature. A hydrophobic barrier pen was used to outline the roots to facilitate reagent application and minimize waste. After drying, PBS was added for 5 min, followed by 30 min of incubation with 2% driselase (SIGMA) in PBS at 37°C. The roots were then washed and permeabilized with 3% IGEPAL CA‐630 (SIGMA) and 10% DMSO (J.T. Baker) for 1 h at room temperature, followed by further washes. Blocking was performed with 3% BSA for 1 h at room temperature, and the roots were incubated overnight at 4°C with a primary antibody against GA3 (Agrisera, AS06194). The next day, roots were washed and incubated with an Alexa Fluor 488‐conjugated secondary antibody for 3 h at 37°C. After additional washing, the roots were mounted in Fluoromount and stored at 4°C in darkness. Imaging was performed using a ZEISS LMS 800 (ZEISS) confocal microscope with a 488 nm laser to excite Alexa Fluor 488; samples were observed using a 20× objective.

### 2.9 Root Sections and Staining for Lignin and Suberin Detection

Treated and control roots of both ecotypes were fragmented into 1 cm segments at the differentiation zones and collected for fixation and sectioning to stain with Basic Fuchsin (BF), Fluorol Yellow 088 (FY), and Renaissance 2200 to visualize lignin, suberin, and cell walls, respectively. The tissue was fixed overnight at 4°C in 4% paraformaldehyde (PFA), then washed and cleared in ClearSee solution for 5 days with daily solution changes. After clearing, the tissue was sequentially stained with 0.2% BF in ClearSee for 1 h, followed by washing and staining with 0.01% FY in ethanol for 30 min, and finally incubated with 0.05% Renaissance for 1 h. In the darkness, the samples were washed and stored in 50% glycerol at 4°C. Cross‐sections of the differentiated root zones were prepared by embedding them in 10% low‐melting agarose; they were sectioned manually and incubated in ClearSee for 24 h before staining with BF, FY, and Renaissance for 15 min each. The stained samples were imaged using a ZEISS LMS 800 confocal microscope, with a 405 nm laser for Renaissance, a 488 nm laser for FY, and a 594 nm laser for BF, using a 20× objective.

### 2.10 Real‐Time Quantitative RT‐PCR Analysis

RNA from roots and shoots of control and treated plants from both ecotypes were isolated as described in Section 2.2. For real‐time RT‐PCR (qRT‐PCR) analysis, 3.5 μg of total RNA was used for cDNA synthesis using SuperScript II (Thermo Fisher Scientific) following the manufacturer's protocol in a 20 μL PCR reaction. All reactions were performed in a BioRad CFX96 using PCR SYBR Green master mix (Thermo Fisher Scientific). The primers used in these experiments are reported in Table S1. Experiments were performed in triplicates for each condition. The efficiency of the oligonucleotides was included in the calculations and normalized with the expression levels using β‐Actin as an endogenous control. Relative expression was calculated using the 2^−ΔCt^ method.

## 3 Results

### 3.1 Transcriptomic Analyses of Calkiní and Acaxochitlán Tomato Plants Reveal Hundreds of Differentially Expressed Genes in Response to Heat Stress

**FIGURE 1:**
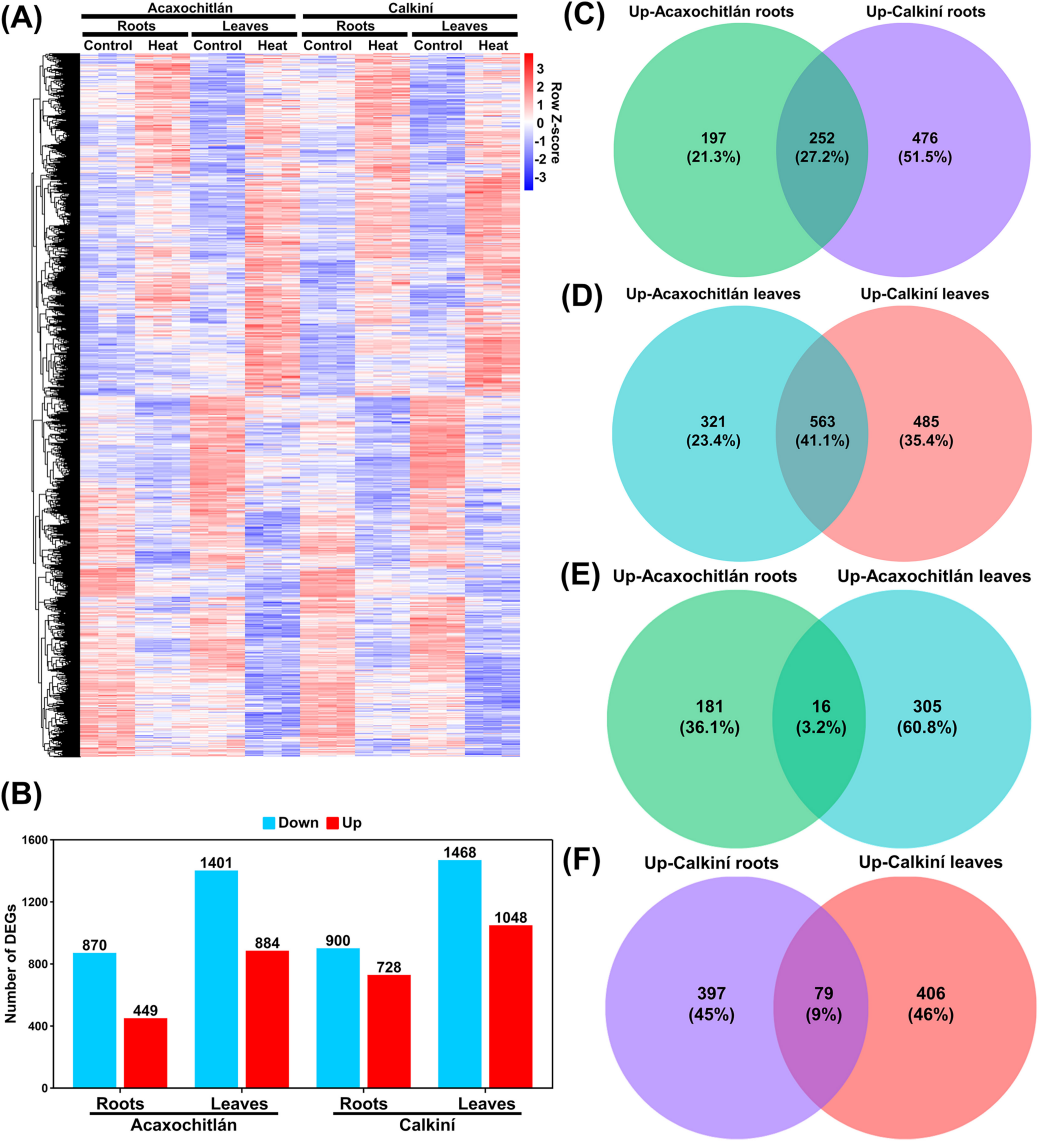
Transcriptomic analyses of Calkiní and Acaxochitlán tissues in response to heat stress. (A) Heatmap of gene expression in the roots and leaves of Acaxochitlán and Calkiní ecotypes under control and heat stress, *n* = 3; red indicates upregulated genes, and blue indicates downregulated genes. (B) Total number of upregulated and downregulated genes in the roots and leaves of both ecotypes in response to heat stress. Venn diagram of common and specific upregulated genes among both ecotypes' roots (C) and leaves (D). Venn diagram showing the overlap of common and specific upregulated genes among the leaves and roots of the ecotype (E) Acaxochitlán and (F) Calkiní.

To unravel the molecular mechanisms involved in response to heat stress in roots and leaves of both tomato ecotypes, we performed RNA sequencing from 12 days old grown seedlings in control conditions and those subjected to heat stress. The reads mapped on the *S. lycopersicum* genome version SL4.0 and were used to analyze global and differential gene expression profiles for control and heat‐treated plants of both ecotypes (Figure 1A; Table S2). A total of 449 DEGs were identified in roots and 884 DEGs in leaves (DEG threshold: *padj* < 0.05; |log2FC| > 2) of ACA plants after 12 days of heat treatment compared to control plants. CAL plants showed more heat‐induced genes, with 728 in roots and 1048 in leaves. The number of DEGs in both ecotypes was more significant in leaves than in roots (Figure 1B).

To know how many up‐regulated genes are shared between ecotypes or how many are specific to one of them, we performed Venn diagram analyses for roots and leaves. Among the 449 up‐regulated genes in ACA roots, only 197 (21.3%) were specific to ACA. Interestingly, the number of up‐regulated genes specific to CAL roots was 476 (51.5%), more than double the number of heat‐induced genes in ACA roots (Figure 1C). Additionally, 321 (23.4%) of the up‐regulated genes in leaves were specific to ACA. In comparison, 485 (35.4%) were specific to CAL.

The DEGs were identified by comparing the same tissues, but different ecotypes were subjected to gene ontology and KEGG analysis. The DEGs identified from comparisons between the same tissue in different ecotypes under heat stress were further classified functionally using gene ontology (GO) and Kyoto Encyclopedia of Genes and Genomes (KEGG) pathway analyses (Figure S1A–D).

The up‐regulated DEGs were annotated to the GO database under the following categories: “biological process,” “molecular function,” and “cellular component.” We show the top 10 enrichments for each GO category (Figure S1A–C). In “biological process,” we found several significally‐enriched terms in roots and leaves of both ecotypes under heat stress, the most enriched terms were: “response to heat,” “protein folding,” “response to temperature stimulus,” “protein maturation,” and “response to abiotic stimulus” (Figure S1A). Furthermore, the GO terms cellular anatomical entity and intracellular anatomical structure cellular component category were enriched in “cellular component” (Figure S1B), and the GO terms “unfolded protein binding,” “protein self‐association,” “heat shock protein binding,” and “ATP‐dependent protein folding chaperone” were significantly enriched in the “molecular function” (Figure S1C). A pathway analysis of the DEGs was conducted using KEGG to detect metabolic pathways in which up‐regulated and down‐regulated genes encoding enzymes were enriched. Under heat stress conditions, pathway analysis revealed the enrichment of protein processing in the endoplasmic reticulum and spliceosome (Figure S1D).

We then wondered how many 197 up‐regulated genes specific to ACA roots are indeed root‐specific or may be shared with the 321 up‐regulated genes in ACA leaves. Venn diagram analyses showed that only 16 up‐regulated genes were shared among ACA tissues. Similar studies for CAL revealed that of 961 up‐regulated genes in roots and leaves, only 79 were shared among tissues (Figure 1E,F).

**FIGURE 2:**
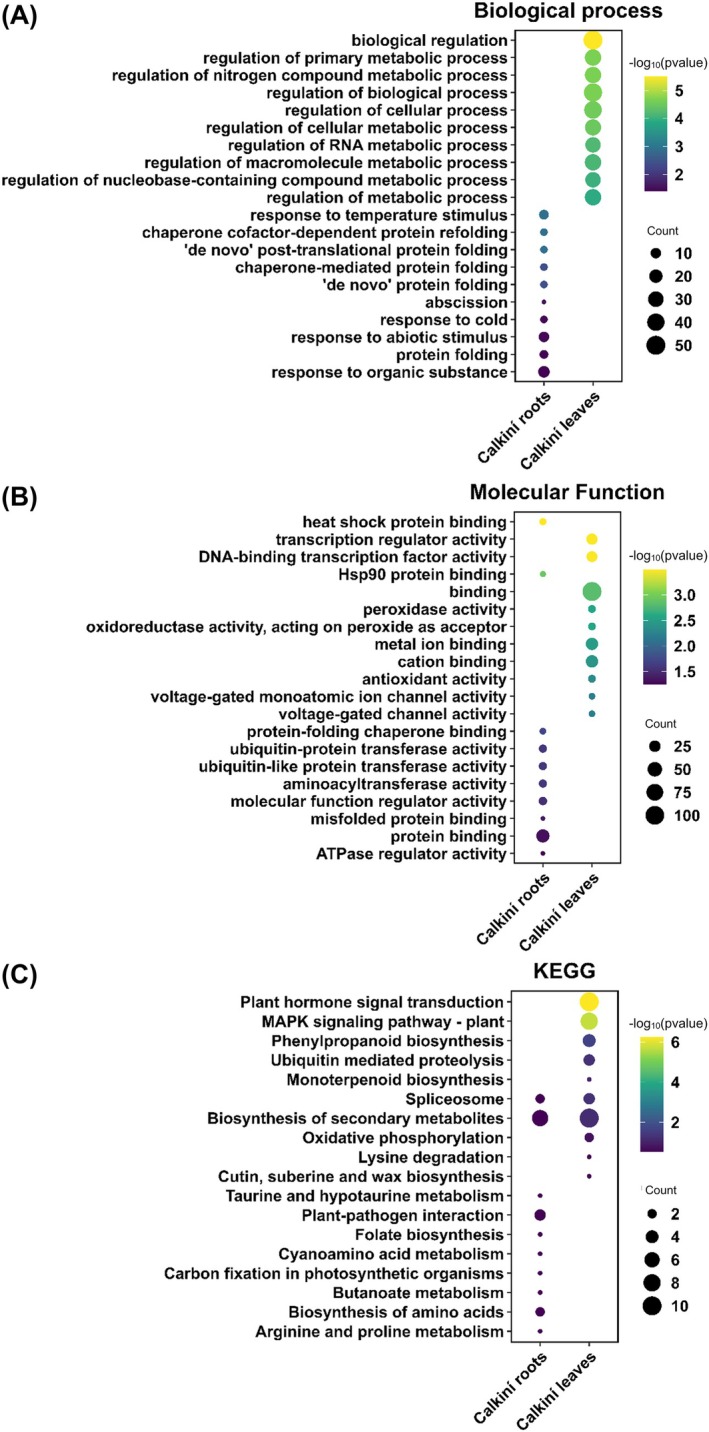
GO and KEGG analysis of Calkiní‐specific up‐regulated genes in roots and leaves. (A) gProfiler biological process, (B) molecular function, and (C) KEGG enriched terms for up‐regulated genes (log(FC) > 2). The 10 terms with lower *p*‐values are shown.

The previous results revealed that the tolerant ecotype CAL displays a robust transcriptional program with more heat‐induced genes than ACA plants. Therefore, we questioned the data for the putative function of such up‐regulated genes that are CAL‐specific. GO (“Biological process” and “molecular function” categories) and KEGG pathway analyses revealed the top 10 enrichment terms in roots and leaves of CAL ecotype. For “biological process” including “biological regulation,” “regulation of primary metabolic process,” and “regulation of nitrogen compound metabolic process” were significantly enriched in CAL leaves. In contrast, terms such as “response to temperature stimulus,” “chaperone cofactor‐dependent protein refolding,” and “‘de novo’ protein folding” were enriched in CAL roots (Figure 2A). For “molecular function” GO terms, the three most enriched terms in CAL leaves were “binding, peroxidase activity” and “oxidoreductase activity,” while for CAL roots the most enriched terms were “heat shock,” “protein binding,” “Hsp90 protein binding,” and “protein‐folding chaperone binding” were enriched (Figure 2B). Additionally, the GO terms “transcription regulator activity” and “DNA‐binding transcription factor activity” were enriched in ACA roots and CAL leaves (Figure S2B). The pathway enrichment analyses using KEGG terms revealed the enrichment of “plant hormone signal transduction,” “MAPK signaling pathway,” and “phenylpropanoid biosynthesis” were enriched in CAL leaves (Figure 2C). In contrast, terms such as “taurine and hypotaurine metabolism,” “plant‐pathogen interaction,” and “folate biosynthesis” were enriched in CAL roots (Figure 2C). Moreover, the “spliceosome” and “biosynthesis of secondary metabolites” terms were significantly enriched in roots and leaves of CAL. These results provided a valuable resource for identifying specific processes and pathways involved in the response to heat stress in CAL ecotype, such as plant hormone signaling, MAPK signaling, phenylpropanoid biosynthesis and heat shock protein binding, that help CAL tolerate heat stress.

### 3.2 Non‐Targeted Metabolomic Analyses Revealed Both Ecotypes' Metabolic Programs Were Induced by Heat Stress

**FIGURE 3:**
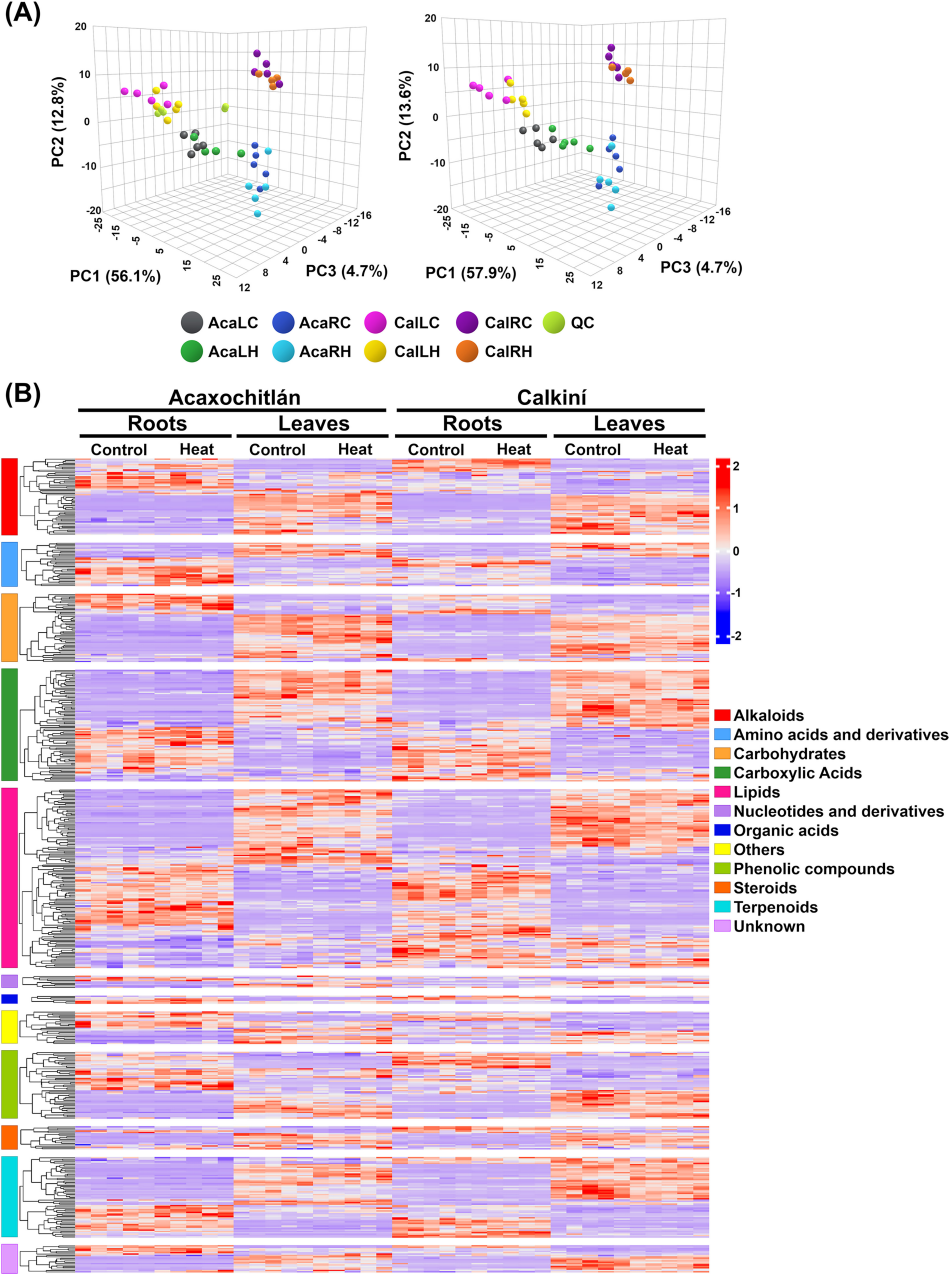
Differential metabolomic analysis of tomato metabolites under heat stress. (A) Principal component analysis (PCA) of metabolites between groups with or without QC as a quality control sample. (B) Cluster heat map of metabolites from the roots and leaves of the Acaxochitlán and Calkiní ecotypes under control and heat stress, *n* = 5; red indicates up‐regulated metabolites and blue indicates down‐regulated metabolites.

To elucidate the metabolic programs displayed in each ecotype and tissue in response to heat stress responses, we compared the differentially accumulated metabolites of roots and leaves from 14‐day‐old plants of both ecotypes. The method for the non‐targeted metabolomics analysis has been used to identify numerous metabolites in tomatoes under single and combined abiotic stresses (Li et al. 2024). The data were analyzed using 3D principal component analysis (PCA). The plot shows consistent clustering of both tissues and ecotypes under heat stress, indicating a stable LC–MS system (Figure 3A). PC1 and PC2 explained more than 60% of the total variation, indicating the significant dissimilarity of metabolites among roots and leaves of both tomato ecotypes under different conditions.

The 509 pre‐identified metabolites (Table S3) in the current study were divided into 12 groups (Figure 3B). The most abundant groups were lipids (131 metabolites), carboxylic acids (82 metabolites), terpenoids (59 metabolites), alkaloids (56 metabolites), and carbohydrates (50 metabolites).

Compared to the controls, 48 and 66 differentially accumulated metabolites (DAMs) were identified in ACA roots and leaves, respectively. While 41 and 57 DAMs were identified in CAL roots and leaves, respectively (DAM threshold: *padj* < 0.05; |log2FC| > 1; Figure S3A). These results suggested that the roots and leaves of both tomato ecotypes under heat stress showed significantly different metabolite accumulation patterns.

Among the obtained DAMs, we performed a KEGG analysis against the annotated canonical pathways to comprehensively investigate the differences in both tomato ecotypes' roots and leaves. The KEGG analysis of tomato DAMs shows that “glycerophospholipid metabolism” is the most enriched category between roots and leaves in both tomato ecotypes. Also, “biosynthesis of various plant secondary metabolites,” “cysteine and methionine metabolism,” “glycine, serine and threonine metabolism,” were shared in the roots and leaves of both ecotypes (Figure S3B).

**FIGURE 4:**
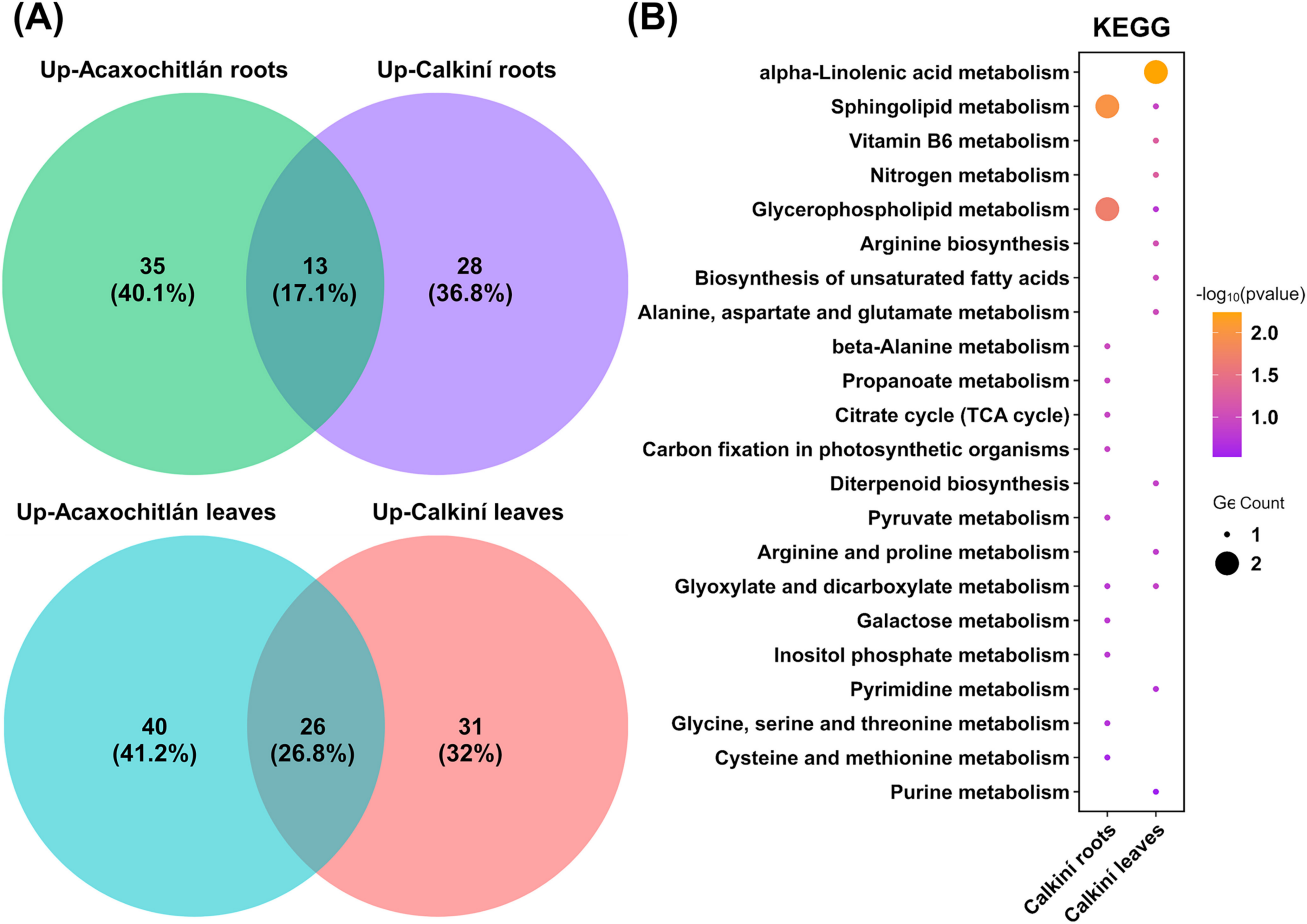
KEGG analysis of tomato up‐regulated metabolites in the Calkiní ecotype under heat stress. (A) Venn diagram showing the overlap of DAMs under heat stress among the roots and leaves of both ecotypes, respectively. (B) KEGG analysis of tomato up‐regulated metabolites under heat stress in the roots and leaves of the Calkiní ecotype. gProfiler KEGG enriched terms for up‐regulated metabolites (log (FC) > 1).

To identify which metabolites are common and shared in the same tissues among CAL and ACA ecotypes, we analyzed our data and displayed them using Venn diagrams (Figure 4A). We identified 28 and 31 metabolites for roots and leaves of the CAL ecotypes under heat stress, respectively. As part of the results, we performed a KEGG analysis (Figure 4B). The most enriched categories, which are shared between tissues, were “sphingolipid metabolism” and “glyoxylate and dicarboxylate metabolism” (Figure 4B). Notably, the DAMs in CAL leaves were enriched in “alpha‐linolenic acid metabolism,” “vitamin B6 metabolism,” “nitrogen metabolism,” “arginine biosynthesis,” and “biosynthesis of unsaturated fatty acids.” Additionally, the DAMs in CAL roots were enriched in “beta‐alanine metabolism,” “propanoate metabolism,” “citrate cycle,” “carbon fixation in photosynthetic organisms,” and “pyruvate metabolism.”

In addition, “glycerophospholipid metabolism” was shared between the roots and leaves of both ecotypes (Figure 4B and Figure S4).

### 3.3 Differential Behavior of Genes and Metabolites Involved in Phenylpropanoids and Suberin Biosynthesis Among Ecotypes

**FIGURE 5:**
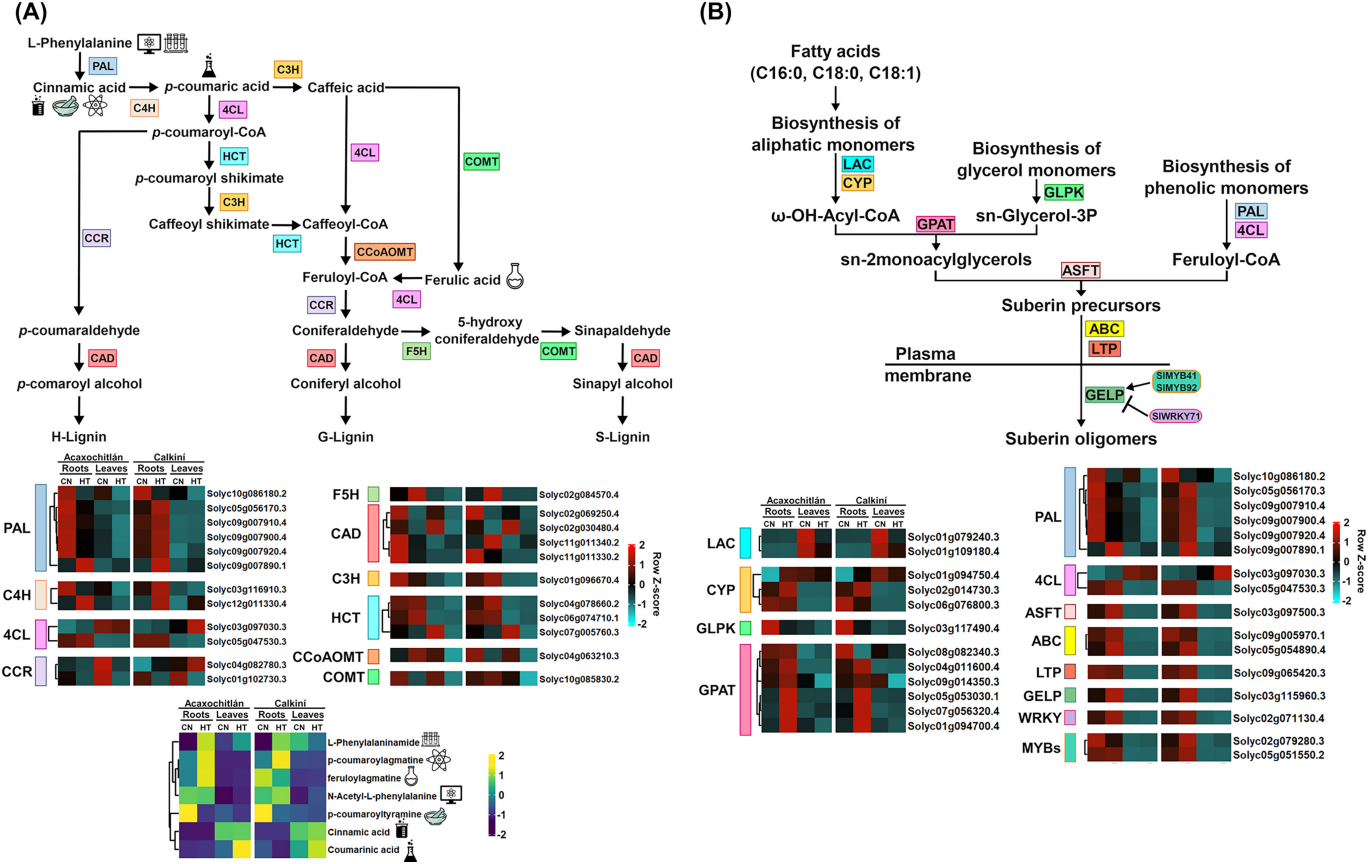
Expression pattern of genes involved in phenylpropanoid biosynthesis and suberin biosynthesis under heat stress in the roots and leaves of both tomato ecotypes. (A) 4CL, 4‐coumarate‐CoA ligase; C3H, p‐coumarate 3‐hydroxylase; C4H, cinnamate 4‐hydroxylase; CAD, cinnamyl alcohol dehydrogenase; CCoAOMT, caffeoyl CoA O‐methyltransferase; CCR, cinnamoyl CoA reductase; COMT, caffeic acid O‐methyltransferase; F5H, ferulate 5‐hydroxylase; HCT, shikimate hydroxycinnamoyl transferase; PAL, phenylalanine ammonia‐lyase. (B) ABC, ATP‐binding cassette transporter; ASFT, aliphatic suberin feruloyl transferase; CYP, cytochrome P450; GELP, GDSL‐type esterase/lipase; GLPK, glycerol kinase; GPAT, glycerol 3‐phosphate acyltransferase; LAC, long‐chain acyl‐CoA synthase; LTP, lipid transfer protein; MYB, myeloblastosis transcription factors; WRKY, WRKY transcription factor.

The combined analysis of transcriptomics and metabolomics data were useful indicators for further in‐depth analyses to identify candidate genes or metabolites. The PaintOmics4 web tool was used to map DEGs and DAMs in KEGG pathways to identify potential molecular mechanisms involved in heat stress response in roots and leaves of both ecotypes. The combined transcriptome and metabolome analysis showed that 23 DEGs and seven DAMs were involved in the phenylpropanoid biosynthesis pathway, and 25 DEGs were involved in suberin biosynthesis in both ecotypes (Figure 5A,B). These results suggest that high‐temperature treatment significantly affected both biosynthetic pathways.

The phenylpropanoid biosynthesis pathway produces lignin, which is made up of three monomers: coumaryl alcohol (H‐lignin), coniferyl alcohol (G‐lignin), and sinapyl alcohol (S‐lignin). Lignin plays a key role in maintaining the cell wall's structural integrity, strength, and rigidity, facilitating water transport, preventing cell wall permeability, and protecting plants from pathogen invasion. The initial three steps of the lignin pathway catalyzed by PAL, C4H, and 4CL provide the basis for all subsequent branches and resulting metabolites (Sun 2020; Meng et al. 2021).

In the initial steps of phenylpropanoid biosynthesis, the key enzyme phenylalanine ammonia‐lyase (PAL) catalyzes the deamination of l‐phenylalanine to produce cinnamic acid. We found that five *SlPALs* are upregulated in CAL roots under heat stress (Figure 5A). Cinnamate 4‐hydroxylase (C4H) hydroxylates cinnamic acid to produce p‐coumaric acid; our results show that the transcripts of two *SlC4Hs* genes (Solyc03g116910.3; Solyc12g011330.4) are upregulated in CAL roots compared to ACA in HT (Figure 5A).

Next, 4‐coumarate‐CoA ligase (4CL) converts p‐coumaric acid, caffeic acid, and ferulic acid into their corresponding CoA derivatives: p‐coumaroyl‐CoA, caffeoyl‐CoA, and feruloyl‐CoA, respectively. The two *Sl4CL* genes show similar expression patterns in the roots of both ecotypes under the same treatments and are upregulated in CAL leaves under high temperature (Figure 5A). During the subsequent steps catalyzed by shikimate hydroxycinnamoyl transferase (HCT), p‐coumarate 3‐hydroxylase (C3H), and ferulate 5‐hydroxylase (F5H), p‐glutaraldehyde is converted to 5‐hydroxy conifer aldehyde, which is important for producing S‐lignin. The *SlF5H* and two *SlHCT* genes show similar expression patterns in the roots of both ecotypes in HT, and the *SlC3H* gene shows an upregulation in CAL roots and down‐regulation in ACA roots in HT (Figure 5A).

Caffeoyl‐CoA O‐methyltransferase (CCoAOMT) and caffeic acid O‐methyltransferase (COMT) catalyze the O‐methylation within this pathway, converting Caffeoyl‐CoA to produce Feruloyl‐CoA and 5‐hydroxyconiferyl alcohol to produce sinapyl alcohol, respectively. The expression of the *SlCCoAOMT* gene was increased in ACA roots, and opposite expression patterns were observed in CAL roots in HT (Figure 5A). In contrast, the *SlCOMT* gene showed an increased expression in CAL roots in HT (Figure 5A).

In the final steps of the phenylpropanoid pathway, CCR converts cinnamoyl‐CoA esters to cinnamaldehydes like p‐coumaraldehyde and sinapaldehyde. One *SlCCR* gene (Solyc04g082780.3) showed increased expression in roots and leaves of the CAL ecotype under heat stress, and the CCR genes showed reduced expression in both tissues of the ACA ecotype in HT (Figure 5A).

Finally, cinnamyl alcohol dehydrogenase (CAD) reduces cinnamaldehydes to cinnamyl alcohols like p‐coumaroyl alcohol, coniferyl alcohol, and sinapyl alcohol as precursors of H‐lignin, G‐lignin, and S‐lignin, respectively. One of the four *SlCAD* genes (Solyc11g011340.2) was strongly induced by heat stress in CAL roots and showed decreased expression patterns in ACA roots in HT (Figure 5A).

Furthermore, we identified seven metabolites involved in lignin biosynthesis. The relative accumulation of four of these metabolites, including feruloylagmatine, l‐phenylalaninamide, N‐acetyl‐l‐phenylalanine, and p‐coumaroylagmatine, increased in the roots of both ecotypes under heat stress (Figure 5A). Additionally, we identified two metabolites, cinnamic acid and coumarinic acid, whose relative accumulation increased in the leaves of both ecotypes under heat stress (Figure 5A).

### 3.4 DEGs Affected by Heat Stress Are Involved in the Suberin Biosynthetic Pathway

Suberin is stored within the interior of the cell wall to develop suberin lamellae, which contain aliphatic monomers, glycerol monomers, and phenolic monomers. In fatty acid metabolism, two *SlLAC* genes and three *SlCYP* genes were identified, whose products catalyze the biosynthesis of aliphatic suberin monomers such as ω‐hydroxy fatty acids (Figure 5B). The *SlLACs* showed a decreased expression in the roots and leaves of both ecotypes under heat stress (Figure 5B). Additionally, two *SlCYPs* (Solyc01g094750.4; Solyc02g014730.3) exhibited increased expression in the roots of both ecotypes under heat stress (Figure 5B).

Regarding the biosynthesis of glycerol monomers, the *SlGLPK* gene was down‐regulated in the roots of both ecotypes under heat stress (Figure 5B). In contrast, the *GPAT* genes, which code for enzymes that also contribute to glycerol backbone formation essential for suberin synthesis, were markedly up‐regulated in the roots of both ACA and CAL ecotypes under heat stress, indicating an alternative route to maintain glycerol availability under these conditions (Figure 5B; Serra and Geldner 2022).

The enzymes encoded by *SlPAL* and *Sl4CL* genes, described in Section 3.3, are involved in the conversion of phenolic monomers to Feruloyl‐CoA (Figure 5B). Ferulic acid is then esterified to ω‐hydroxy‐acids and primary alcohols by the feruloyl transferase ASFT, a key step in suberin assembly. Our results show that the *ASFT* gene is up‐regulated in the roots of both ecotypes under heat stress, suggesting the continued incorporation of ferulic acid into the suberin polymer under heat stress (Figure 5B; Serra and Geldner 2022).

Related to the previously‐described result, it is important to mention that many of the enzymes involved in the synthesis of suberin monomers are located within the endoplasmic reticulum (ER), the assembly of suberin lamellae occurs at the plasma membrane and within the cell wall (Serra and Geldner 2022; Chen et al. 2025). The transport of suberin monomers from the ER to the plasma membrane and cell wall is mediated by ABC transporters and lipid transfer proteins (LTPs); therefore, we speculate that, under heat stress, there is an active delivery of suberin monomers which polymerize and integrate into the cell wall structure in order to face the stress (Figure 5B; Xin and Herburger 2021; Woolfson et al. 2022). Interestingly, the *SlABC* and *SlLTP* genes displayed similar expression patterns in the roots of both ecotypes under control and heat stress conditions, suggesting that these genes were not affected by the heat stress (Figure 5B). Finally, acyl‐glycerol‐esters are transported into the apoplast, where they serve as substrates for polymerization by GELP enzymes, which mediate the final cross‐linking steps in suberin formation. The expression of the *SlGELP* gene increases in the roots of both ecotypes under heat stress, indicating active suberin polymer assembly under stress conditions (Figure 5B).

Previous studies have reported that *SlMYB92* (Solyc05g051550.2) and *SlMYB41* (Solyc02g079280.3) are positive regulators of suberin biosynthesis in the tomato root exodermis, while *SlWRKY71* (Solyc02g071130.3) functions as a repressor of suberin biosynthesis genes (Jo et al. 2025). Our transcriptomic results show up‐regulation of *SlMYB92*, *SlMYB41*, and *SlWRKY71* in CAL roots under heat stress (Figure 5B). In ACA roots, we also observed the up‐regulation of *SlMYB92* and *SlWRKY71* and the down‐regulation of *SlMYB41* (Figure 5B). These findings suggest that ACA and CAL regulate suberin biosynthesis differently under heat stress, which may affect their suberin levels and heat tolerance.

### 3.5 Calkiní Plants Exhibit Enhanced Suberin Deposition in the Exodermis and Endodermis of the Differentiation Zone of Roots Under Heat Stress

**FIGURE 6:**
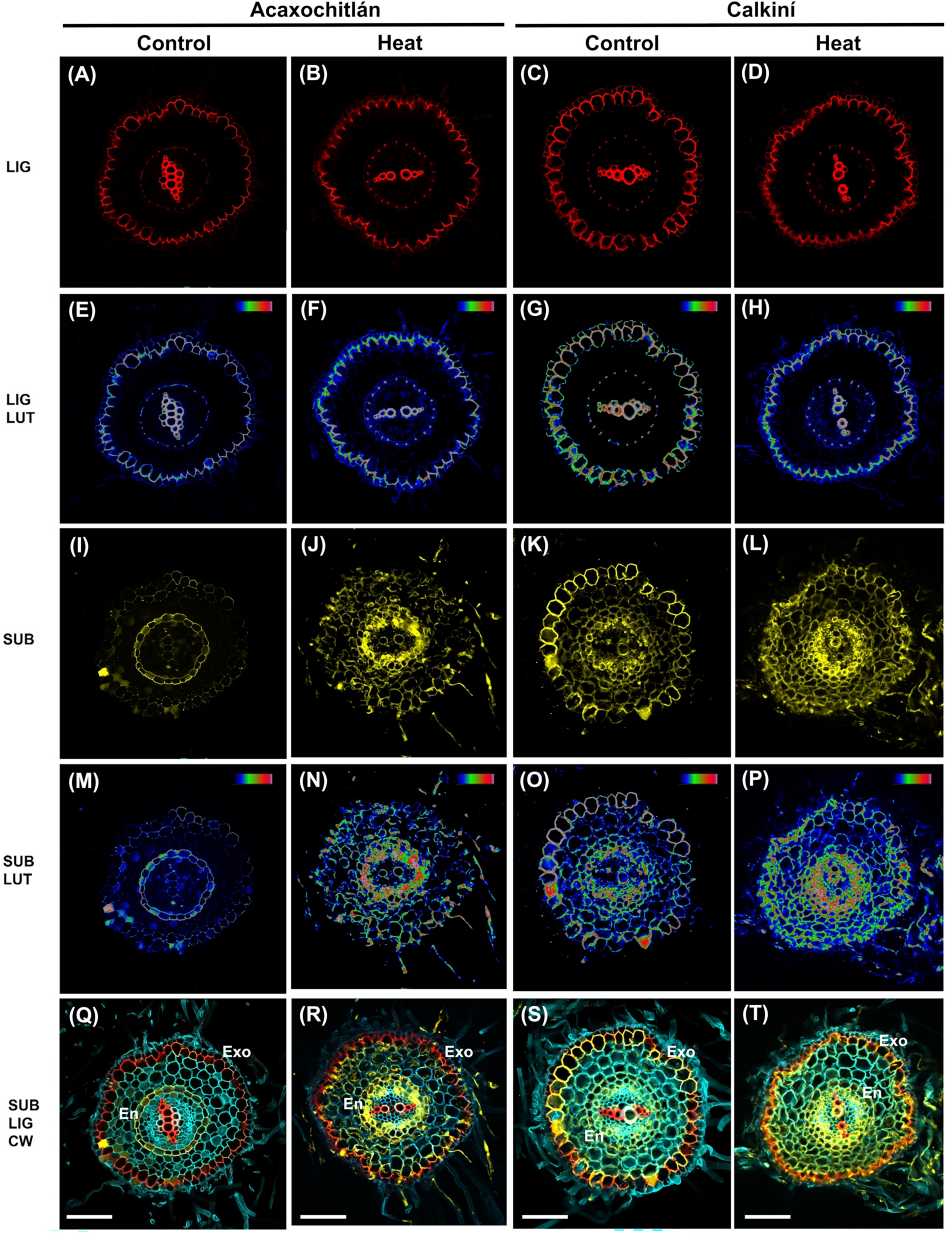
Lignified, suberised and secondary cell walls in hand‐cut transverse sections of differentiated root tissue of Acaxochitlán and Calkiní ecotypes under control and heat stress, visualized by confocal microscopy (Zeiss LMS 800). (A–D) Basic Fuchsin staining showing lignified endodermis (594 nm excitation), and the respective Look Up Table (LUT) patterns (E–H). (I–L) Fluorol yellow staining showing suberised endodermis and exodermis (488 nm excitation) and the respective Look Up Table (LUT) patterns (M–P). Size bars = 100 μm.

Since our integrated approach revealed a differential behavior among ecotypes in DEGs and DAMs associated with suberin production, we obtained hand‐cut transverse sections of differentiated root tissue of control and treated plants of both ecotypes. To specifically visualize lignin and suberin deposition, we performed triple staining using Basic Fuchsin (BF), Fluorol Yellow 088 (FY), and Renaissance 2200 to visualize lignin, suberin, and cell walls, respectively, as described by Sexauer et al. (2021). Roots of both ecotypes under control and heat stress treatment displayed lignin deposition in the vascular tissue, endodermal Casparian strips, and the exodermis (Figure 6A–D). We observed no significant changes in lignin deposition under either control or heat stress conditions in both ecotypes (Figure 6A–D). Additionally, when we applied the Look‐Up Table (LUT, a tool that maps numerical pixel values to specific colors for display), we confirmed no changes in the lignin deposition (Figure 6E–H).

Interestingly, we observed differences between treatments and ecotypes, such as a weak suberin deposition in the endodermis and exodermis of ACA roots under control conditions, while under heat stress, ACA roots showed suberin deposition in the endodermis (Figure 6I,J). On the other hand, CAL control roots exhibited already more suberin deposition in both the endodermis and exodermis when compared to controls of ACA (Figure 6I–K). A dramatic increase in suberin deposition was observed in endodermal cells of CAL roots under heat stress (Figure 6K,L). When we applied the LUT scale, we further observed higher suberin deposition in CAL roots under heat stress in the endodermis, exodermis, and other cell layers (Figure 6M–T). The merge of the triple staining summarizes the clear differences in suberin deposition among treatments and ecotypes (Figure 6Q–T).

### 3.6 Gibberellins Are Differentially Accumulated Among Treatments and Ecotypes in the Root Tips

**FIGURE 7:**
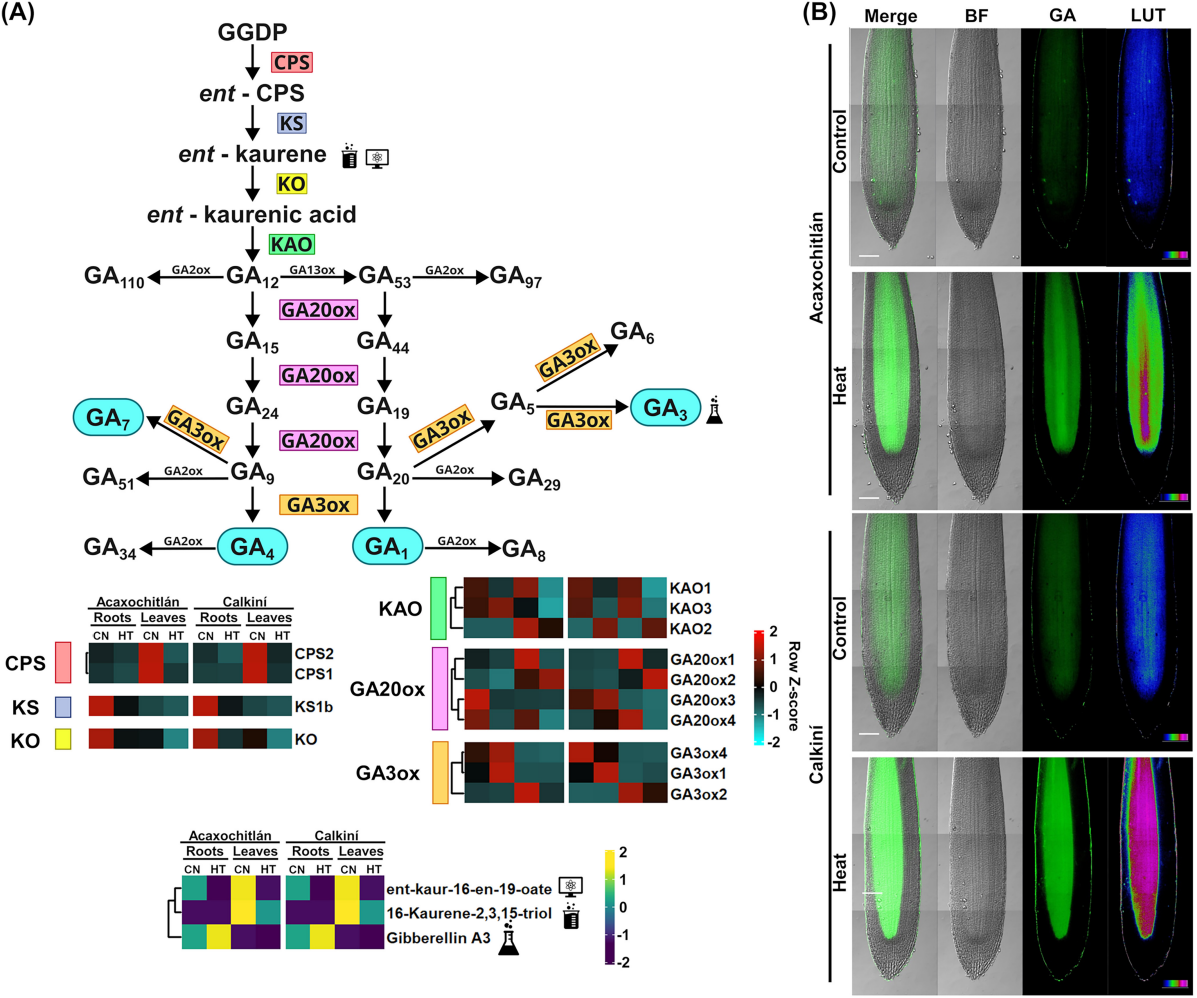
Heat stress significantly increases gibberellin (GA3) accumulation in the meristematic zones of Calkiní roots. (A) Expression profiles of genes and metabolites involved in the gibberellin biosynthesis pathway. CPS, ent‐copalyl diphosphate synthase; GA20ox, GA20 oxidase; GA3ox, GA3 oxidase; GGDP, geranylgeranyl diphosphate; KAO, ent‐kaurenoic acid oxidase; KO, ent‐kaurene oxidase; KS, ent‐kaurene synthase. (B) Fluorescence microscopy of gibberellin gradients in the meristematic zones of roots in the Acaxochitlán and Calkiní ecotypes under control and heat stress treatments. Scale bars = 100 μm.

We further investigated the expression of GA metabolism genes in response to heat stress. GAs are diterpenoids produced from the general substrate geranylgeranyl diphosphate (GGPP), which is converted to ent‐kaurene by ent‐copalyl diphosphate synthase (CPS) and ent‐kaurene synthase (KS). The results showed that the expression of early GA synthesis genes, including *SlCPS* genes and *SlKS* genes, was down‐regulated under heat stress in the roots and leaves of both CAL and ACA ecotypes (Figure 7A). Subsequently, ent‐kaurene is converted to ent‐kaurenic acid by ent‐kaurene oxidase (KO), and the *SlKO* gene was down‐regulated under heat stress in the roots and leaves of both ecotypes (Figure 7A).

Additionally, the *SlKAO1* gene, which encodes for the enzyme that converts ent‐kaurenic acid to GA_12_, was up‐regulated in the roots and leaves of the CAL ecotype under heat stress (Figure 7A). In contrast, *SlKAO2* was up‐regulated in the roots of the ACA ecotype (Figure 7A). Following this pathway, GA_12_ is processed by GA20ox and GA3ox enzymes in two separate branches to produce bioactive GAs. The non‐13‐hydroxylation branch yields GA_7_ and GA_4_, while the 13‐hydroxylation branch yields GA_3_ and GA_1_ as bioactive GAs (Katyayini et al. 2020). We observed that the expression of *SlGA20ox* and *SlGA3ox* genes, which are critical for the production of bioactive GAs from GA12, was differentially regulated under heat stress, indicating that heat conditions may modulate these metabolic steps and impact the pool of bioactive GAs in both ecotypes (Figure 7A).

To further dissect these branch‐specific responses, we analyzed the tRNA‐seq profiles of *SlGA20ox* and *SlGA3ox* genes under heat stress in both ecotypes. The *SlGA20ox* genes exhibited diverse responses to heat stress in both ecotypes (Figure 7A). In the roots of CAL, *SlGA20ox3* and *SlGA20ox4* showed increased expression under heat stress, while in the roots of ACA, the expression of all *SlGA20ox* genes decreased (Figure 7A). Finally, the *SlGA3ox1* gene showed increased expression in the roots of both ecotypes, while *SlGA3ox2* remained unaffected by heat stress in the roots of both ecotypes (Figure 7A).

Furthermore, we identified in our metabolomic analyses ent‐kaur‐16‐en‐19‐oate and 16‐Kaurene‐2,3,15‐triol in the early steps of GAs biosynthesis (Figure 7A). These metabolites belong to the class of organic compounds known as kaurane diterpenoids, and their relative accumulation was consistent with the RNA‐seq profiles in roots of both ecotypes under heat stress (Figure 7A,B). Additionally, we detected the relative accumulation of GA_3_ in the roots of both ecotypes under heat stress, and these results were consistent with the expression profiles of *SlGA3ox1* (Figure 7A), which has been previously reported as the major supplier of the GA3ox enzyme in Arabidopsis and responsible for the synthesis of bioactive GAs (Figure 7A; Mitchum et al. 2006; Sun 2008).

To corroborate the differential GA3 distribution among ecotypes in situ, we performed immunohistofluorescence assays in the control roots and treated 14‐day‐old roots of both ecotypes (Figure 7B). We observed that GA3 is strongly accumulated in ACA and CAL roots in response to heat stress, but that accumulation was higher in the roots of CAL plants (Figure 7B). This result is consistent with previous reports, where the accumulation of gibberellin is induced by abiotic stress in plants (Shohat et al. 2021; Guo et al. 2022; Wang, Luo, et al. 2023).

### 3.7 Differential PA Accumulation and GAPC Expression Among Ecotypes in Response to Heat Stress

**FIGURE 8:**
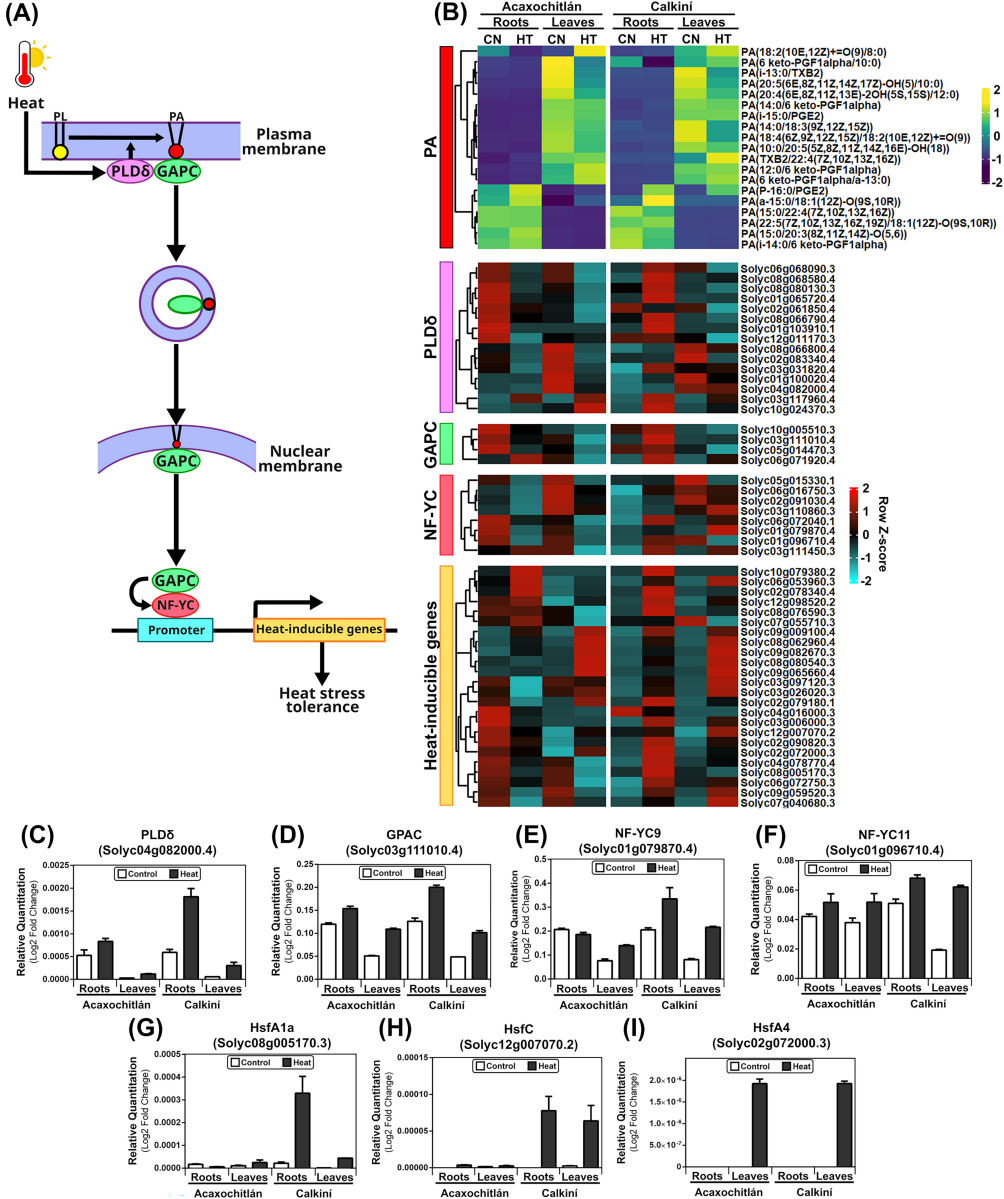
Metabolites and genes involved in the PA‐GAPC‐NFY heat stress tolerance pathway are differentially accumulated and expressed among ecotypes. (A) A proposed model for PLDδ‐derived PA interacting with GAPC and the co‐movement of PA‐GAPC into the nucleus in response to heat stress in Arabidopsis. (B) Heat map of expression profiles of identified PAs in the metabolome data, using a Viridis color scale. Heat map of expression profiles of transcriptome data for orthologous genes involved in the heat stress response, utilizing a cyan‐to‐red color scale. (C) qRT‐PCR analysis of seven genes involved in the heat stress response in tomatoes. GAPC, cytosolic glyceraldehyde‐3‐phosphate dehydrogenase; HSF, heat shock transcription factor; NF‐YC, nuclear factor Y subunit C; PA, phosphatidic acid; PLDδ, phospholipase Dδ.

Our metabolomic approach showed an enriched accumulation of lipids, including phosphatidic acid or PA (Figure 3B). Previous studies in Arabidopsis showed that, in response to heat stress, plasma membrane‐associated phospholipase Dδ (PLDδ) and its product phosphatidic acid (PA) mediate nuclear translocation of glyceraldehyde‐3‐phosphate dehydrogenase (GAPC) through lipid‐protein interaction and vesicle trafficking (Kim et al. 2020, 2022; Yao et al. 2024). Nuclear accumulation of GAPC can interact with the NF‐YC transcription factor, enhancing the expression of heat‐inducible genes and promoting heat tolerance in Arabidopsis (Figure 8A; Kim et al. 2020, 2022; Yao et al. 2024).

Considering the accumulation of lipids in plants subjected to heat stress (cita la figura correspondiente) and the model described in Figure 8, panel A, we analyzed the accumulation patterns of PAs in our metabolome data, as well as the transcriptional behavior of genes orthologues to *PLDδ*, *GAPC*, *NF‐YC*, and *HSF* in our RNA‐seq data in the roots and leaves of both ecotypes under control and heat stress conditions. We identified 19 types of PA in our metabolome data, of which five were increased in the leaves of CAL and four of them were increased in the leaves of ACA under heat stress (Figure 8B). Additionally, three PAs were increased in the roots of CAL and ACA ecotypes under heat stress (Figure 8B). Moreover, in our transcriptome data, we identified 15 genes that code for PLDδ, of which 10 showed an increased expression in the roots of CAL under heat stress (Figure 8B). In contrast, only one showed an increase in the roots of ACA under the same conditions (Figure 8B). Furthermore, we found four genes that code for GAPCs, and interestingly, these genes were up‐regulated in the roots of CAL, with only one being up‐regulated in the roots of ACA under heat stress (Figure 8B). Additionally, this gene was also up‐regulated in the leaves of CAL. It has been shown that when GAPC localizes in the nucleus, it interacts with the NF‐YC transcription factors (Figure 8A; Kim et al. 2020, 2022; Yao et al. 2024). We identified eight genes that encode for NF‐YCs in tomato and observed an increased expression of four of those genes in the roots and leaves of CAL under heat stress. On the other hand, only one *NF‐YC* gene was up‐regulated in the roots of ACA, with expression in the leaves decreasing under heat stress (Figure 8B).

The direct interaction between GAPC and NF‐YC enhances the expression of heat‐inducible genes, which include heat shock transcription factors (HSFs; Kim et al. 2020). In *S. lycopersicum* , 26 *HSF* genes have been reported (Yang et al. 2016), of which we identified 24 in our transcriptomic data (Figure 8B). We observed that 13 HSF genes were up‐regulated in the roots of CAL, while six were up‐regulated in the roots of ACA under heat stress (Figure 8B).

Based on the RNA‐seq expression profile analysis, we selected seven key genes involved in the heat stress tolerance pathway, and their expression was further evaluated by quantitative real‐time PCR. The transcripts of the diverse genes analyzed in Figure 8C displayed a range of expression patterns during heat stress, and most of the changes in transcript accumulation were consistent with those observed in the RNA‐seq profiles. The expression profiles of *SlPLDδ* (Solyc04g082000.4), a gene previously reported to increase root length through its PLDδ activity (Wang, Zhang, et al. 2023), showed increased relative expression in the roots of CAL ecotype under heat stress (Figure 8C). The expression of *SlGPAC* (Solyc03g111010.4) showed increased expression in the roots and leaves of both ecotypes under heat stress, but the relative quantitation was higher in CAL roots (Figure 8D). *SlNF‐YC9* (Solyc01g079870.4) and *SlNF‐YC11* (Solyc01g096710.4), previously reported to be up‐regulated during fruit ripening (Li et al. 2016), showed increased expression in CAL roots under heat stress (Figure 8E,F). Two important HSF showed increased expression in CAL roots (Figure 8G,H), *SlHsfA1a* (Solyc08g005170.3) which acts as a master regulator in heat stress responses in tomatoes, and *SlHsfC* (Solyc12g007070.2) is reported to play a role in abiotic stress and plant thermotolerance (Graci and Barone 2023). Another well‐described tomato HSF, *SlHsfA4* (Solyc02g072000.3; Baniwal et al. 2007), was expressed only in the leaves of both CAL and ACA ecotypes under heat stress (Figure 8I).

## 4 Discussion

In this work, we applied a combination of “omics” to obtain and analyze the molecular response to heat stress of two heirloom tomato ecotypes, one that grows in high temperatures (Calkiní, CAL) and one that grows in low temperatures (Acaxochitlán, ACA). Although both ecotypes display a common set of transcripts and metabolites in response to heat stress, there are also specific responses in terms of metabolites and transcripts in the heat‐tolerant ecotype.

One of the significant findings in this work is that the roots of the CAL ecotype exhibit a substantial accumulation of suberin in the exodermis and endodermis. However, the differences in phenylpropanoid accumulation in the exodermis, endodermis, and vascular tissue (comprising the xylem and phloem) remain unchanged, regardless of control or heat stress conditions, in the roots of the CAL and ACA ecotypes. Under abiotic stress, the phenylpropanoid pathway is activated, increasing the synthesis of phenolic compounds, such as flavonoids and phenolic acids, which help scavenge reactive oxygen species (ROS) and protect plant cells from oxidative damage. The activation of the phenylpropanoid pathway under heat‐stress conditions is supported by the higher transcript level genes coding for enzymes involved in phenolic compound biosynthesis, such as phenylalanine ammonia‐lyase (PAL), cinnamate 4‐hydroxylase (C4H), and 4‐coumarate‐CoA ligase (4CL), observed under heat stress in the roots of both ecotypes, aiding in stress mitigation. Overall, the enhanced production of phenylpropanoids improves plant resilience and adaptability to environmental challenges (Rigano et al. 2016; Sharma et al. 2019; Ray et al. 2024). It has previously been indicated that heat stress at 35°C in tomato plants increases the production of phenolic compounds, including phenylpropanoids, with increased PAL activity under heat stress. In contrast, the activity of peroxidase (POD) and polyphenol oxidase (PPO) decreases. This is considered a response mechanism to thermal stress, aiding the plant's acclimation strategy (Rivero et al. 2001). Moreover, as previously reported by Paupière et al. (2020), no significant alterations were observed in the levels of phenolics in WT plants subjected to heat stress (1 h, 38°C), suggesting that phenolic compounds may not respond to heat stress in the same way as other metabolites. These findings are like our lignin staining results, where no significant changes in phenylpropanoid accumulation under heat stress were observed in transverse sections of differentiated root tissue (Figure 6A–D). Our study suggests this pathway may not play a significant role in root tissue responses to heat stress, emphasizing the variability of stress response mechanisms across tissues and conditions.

Suberin abundance varies according to plant and tissue types, developmental stage, and the plant's ability to respond to environmental changes. Suberin is a complex lipophilic macromolecule that plays a key role in plant cell walls by preventing water loss and enhancing water retention within root systems. Composed of long‐chain fatty acids (suberin acids) and glycerol, suberin forms a polyester (Graça 2015; Harman‐Ware et al. 2021). Previously, it has been demonstrated in Arabidopsis roots that suberin deposition in the endodermis occurs after the synthesis and deposition of the Casparian strip (Naseer et al. 2012; Serra and Geldner 2022). Additionally, Cantó‐Pastor et al. (2024), Jo et al. (2025), and Manzano et al. (2025) demonstrated suberin deposition in the exodermis of roots in *S. lycopersicum* . Building on these findings in tomatoes, we observed a suberin accumulation in the exodermis that was similar to what was reported by Cantó‐Pastor et al. (2024). However, we also identified suberin accumulation in the endodermis of roots under heat stress, suggesting it may act as a tolerance mechanism in response to heat stress in the CAL ecotype (Figure 6I–L). The dual deposition of suberin in the exodermis and endodermis under heat stress in CAL roots may provide a physical and biochemical barrier, enhancing root resilience to elevated temperatures. This adaptation could help maintain water balance and protect against stress‐induced damage, which is crucial for plant survival in heat‐stressed environments.

While our study provides valuable insights, there are limitations to consider. For example, phenylpropanoid accumulation may vary over time, and our study captures a single snapshot under heat stress. Additionally, our observations were limited to root tissues; other plant organs may show different responses.

To further elucidate the role of suberin and phenylpropanoids in heat stress adaptation, we recommend conducting time‐course experiments to monitor phenylpropanoid and suberin dynamics throughout the stress period, using genetic tools such as knockout or overexpression lines of suberin‐related genes to confirm their role in heat stress tolerance, and expanding the study to include leaves and stems to provide a comprehensive view of plant‐wide responses to heat stress.

Another interesting finding in our ecotypes is that we identified the gibberellin accumulation in the meristematic zone of CAL roots under heat stress (Figure 7B). GA is crucial for regulating the root structure and function in response to developmental and environmental cues. As previously reported, gibberellin is required for root suberization (Binenbaum et al. 2023). Gibberellin (GA) is key in regulating plant development, particularly in forming suberin in the endodermis, a barrier in plant roots that aids in water and nutrient regulation. It interacts with other hormones, especially abscisic acid (ABA), to coordinate root suberization, which is essential for proper endodermal differentiation. Gibberellin accumulates in elongating endodermal cells, promoting their differentiation and suberin formation, highlighting complex hormonal interactions that influence root development and environmental adaptations (Ubeda‐Tomás et al. 2008; Binenbaum et al. 2018; Wang et al. 2020).

Gibberellin biosynthesis and transport between roots and shoots is crucial for a coordinated development in tomato plants. Grafting experiments with GA‐deficient mutants demonstrated that restoring GA levels can recover growth features, indicating that GA movement and concentration directly influence root and shoot development (Omena‐Garcia et al. 2025).

They also play a key role in helping plants cope with heat stress by promoting seed germination, seedling growth, and flowering even under high temperatures. This thermotolerance is mediated, in part, by the regulation of gibberellin levels, which control important pathways. These include the breakdown of DELLA proteins, which are negative regulators of GA signaling and activators of stress‐related genes. Together, these mechanisms allow plants to adapt and maintain growth during periods of heat stress (Kumar et al. 2012; Srivastava and Pandey 2023).

Furthermore, genes involved in stress responses, such as heat‐shock proteins (HSPs), are part of the regulatory networks active during somatic embryogenesis. This suggests a possible link between stress adaptation processes and hormonal regulation, including the involvement of gibberellins (Valencia‐Lozano et al. 2024).

These findings significantly impact our understanding of how plants adapt their root structures to environmental stresses. The increased accumulation of GA in the meristematic zone under heat stress indicates that GA may serve as a signaling mechanism to trigger protective root modifications such as suberization to regulate water and nutrient transport under adverse conditions. While our findings highlight the role of GA in heat stress responses, there are limitations to consider. First, the exact molecular mechanisms linking GA accumulation to suberin biosynthesis under heat stress remain unclear. The results are specific to the CAL tomato ecotype, and the generalizability of these findings to other plant species or ecotypes requires additional investigation. To build upon this work, future studies should focus on elucidating the precise molecular pathways by which GA regulates suberization under heat stress, providing a more comprehensive understanding of root adaptations to environmental stresses. Additionally, these insights could inform breeding or genetic engineering strategies to enhance plant resilience to heat stress.

Our metabolomic approach identified phosphatidic acid (PA) accumulation in leaves and roots under heat stress in both ecotypes. This led us to investigate the transcriptional behavior of key genes involved in the PA‐GPAC‐NFYC pathway reported by Kim et al. (2020, 2022) and Yao et al. (2024) (Figure 8A) and we analyzed *PLDδ, GPAC, NF‐YC*, and heat shock transcription factors. Using qRT‐PCR, we confirmed that these genes were significantly up‐regulated in CAL roots under heat stress, highlighting their potential role in heat stress adaptation.

Phospholipase Ds (PLDs) are key phospholipid hydrolases that catalyze the hydrolysis of phospholipids to produce phosphatidic acid (PA), a signaling molecule critical for stress responses. Among the PLD family, PLDδ has been reported to respond to various stressors. For example, Guo et al. (2024) showed that *PLDδ* expression increased in tomato leaves under cold stress (4°C) but decreased under salt stress and drought conditions. Our results extend these findings, demonstrating that *SlPLDδ* transcript levels are up‐regulated in CAL roots under heat stress (Figure 8B). Additionally, in response to heat stress, Glyceraldehyde‐3‐phosphate dehydrogenase (GAPDH) translocates from the cytoplasm to the nucleus, where it interacts with the NF‐YC transcription factor to enhance heat tolerance and regulate the expression of heat‐inducible genes (Kim et al. 2020, 2022; Yao et al. 2024). Consistent with these findings, our results demonstrated that *SlGAPC* transcript levels are significantly up‐regulated in CAL roots under heat stress.

The nuclear Factor Y (NF‐Y) TF family, which is involved in regulating plant growth, development, and abiotic and biotic stress responses, is categorized as NF‐YA, NF‐YB, and NF‐YC proteins (Li et al. 2008, 2016). Previously, NF‐YC9 has been reported to be up‐regulated during ripening and induced by ethylene in tomatoes. It was also up‐regulated in CAL roots under heat stress.

Finally, in tomatoes, HsfA1a (Solyc08g005170.3) has been reported to act as a master regulator and is expressed in specific tissues such as roots, leaves, and mature green fruits (Yang et al. 2016; El‐shershaby et al. 2019; Graci and Barone 2023). Through qRT‐PCR analysis, we identified the up‐regulation of HsfA1a in CAL roots under heat stress compared to the ACA ecotype (Figure 8C). HsfA1a is expressed at low levels in the roots and leaves of ACA under both conditions. At the same time, its higher expression is limited to CAL roots, which may suggest a role in regulating heat stress response and tolerance.

Despite identifying the up‐regulation of these genes and the PA accumulation under heat stress, the roles of these genes in heat stress adaptation have not been fully characterized at the molecular or physiological levels. Functional studies (e.g., gene knockout and overexpression experiments) should be conducted to confirm the specific contributions to heat stress responses in these ecotypes and further elucidate the roles of these genes in heat stress tolerance.

In summary, there are consistent differences in the transcriptional and metabolic programs between the two heirloom tomato ecotypes studied, which explain the Calkini ecotype's neat stress tolerance and its capacity to thrive upon high temperatures in Maya‐land.

## Author Contributions

A.C.‐R. and J.J.O.‐O. conceived and designed the research. L.F.M.‐L. conducted plant heat stress experiments and transcriptomic and metabolomic analyses. A.E.‐C. performed qRT‐PCR analyses, lignin and suberin staining, and immunofluorescence assay. L.F.M.‐L. wrote the first draft of the manuscript with critical feedback from all co‐authors. All authors approve the submitted version.

## Supporting information


**Data S1:** Supplementary Figures.

**Data S2:** Supplementary Tables.

## Data Availability

The RNA‐Seq data underlying this article are available in the Sequence Read Archive (SRA) of the National Center for Biotechnology Information (NCBI) at http://www.ncbi.nlm.nih.gov/bioproject/1283287 or under the BioProject accession (PRJNA1283287).
