# Supplementary material for: Multi‐Omics Analyses on Maya‐Land Tomatoes Shed Light on Plant Strategies to Thrive in High Temperatures
Source: Physiol Plant. 2025 Jul 27;177(4):e70429. doi: 10.1111/ppl.70429 (PMC12301587; doi:10.1111/ppl.70429)
Supplement: Supplementary file 1 — Data S1: Supplementary Figures. [file PPL-177-e70429-s001.pdf]

Supplementary Data

Multi-omics analyses on Maya-land tomatoes shed light on plant strategies to thrive in high temperatures

Luis F. Maceda-López[1,2], Annie Espinal-Centeno[2], Jose Juan Ordaz-Ortiz[1]* and Alfredo Cruz-Ramírez[2]*

[1]Metabolomics and Mass Spectrometry Laboratory, Unidad de Genómica Avanzada, Centro de Investigación y Estudios Avanzados, Instituto Politécnico Nacional, Irapuato 36824, Guanajuato, México.

[2]Molecular and Developmental Complexity Group, Unidad de Genómica Avanzada, Centro de Investigación y Estudios Avanzados, Instituto Politécnico Nacional, Irapuato 36824, Guanajuato, México.

*Correspondence: alfredo.cruz@cinvestav.mx, jose.ordaz.ortiz@cinvestav.mx

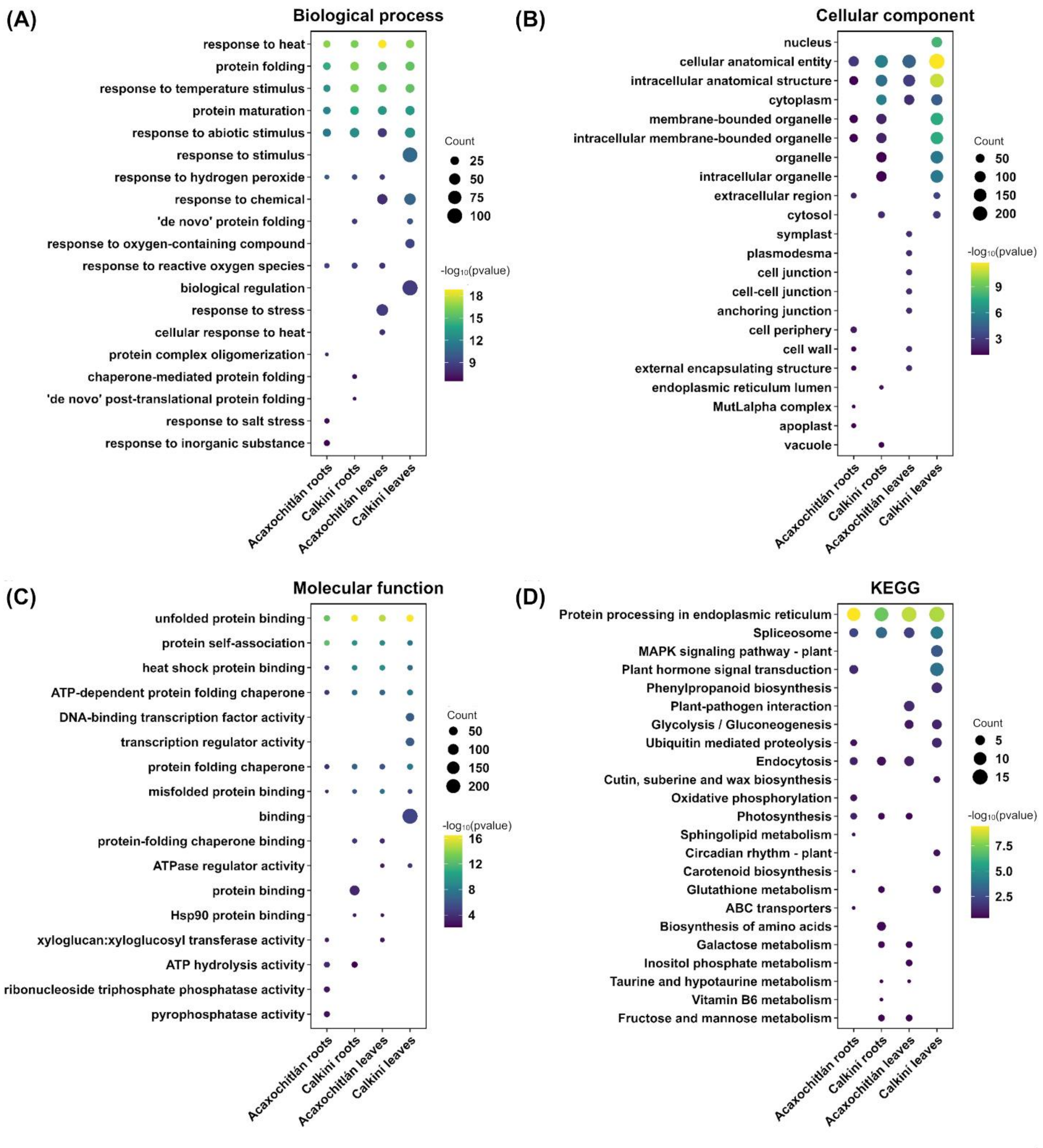

Figure S1 GO, and KEGG analysis of tomato up-regulated genes under heat stress in roots and leaves of Acaxochitlán and Calkiní ecotype under heat stress based on transcriptomics. GProfiler (A) biological process, (B) cellular component, (C) molecular function and (D) KEGG enriched terms for up-regulated genes (log(FC) > 2). The 10 terms with lower p-values are shown.

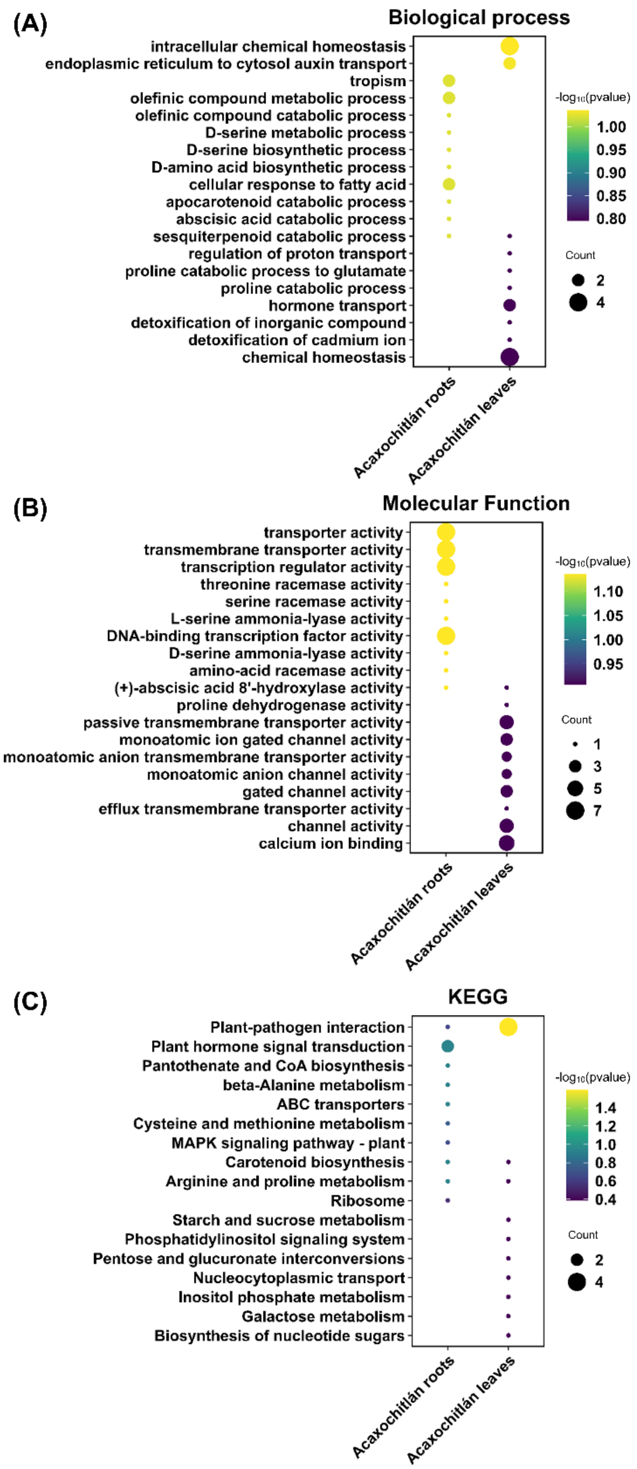

Figure S2 GO and KEGG analysis of specific up-regulated genes under heat stress in roots and leaves of Acaxochitlán plants. gProfiler (A) biological process, (B) molecular function and (D) KEGG enriched terms for up-regulated genes (log(FC) > 2). The 10 terms with lower p-values are shown.

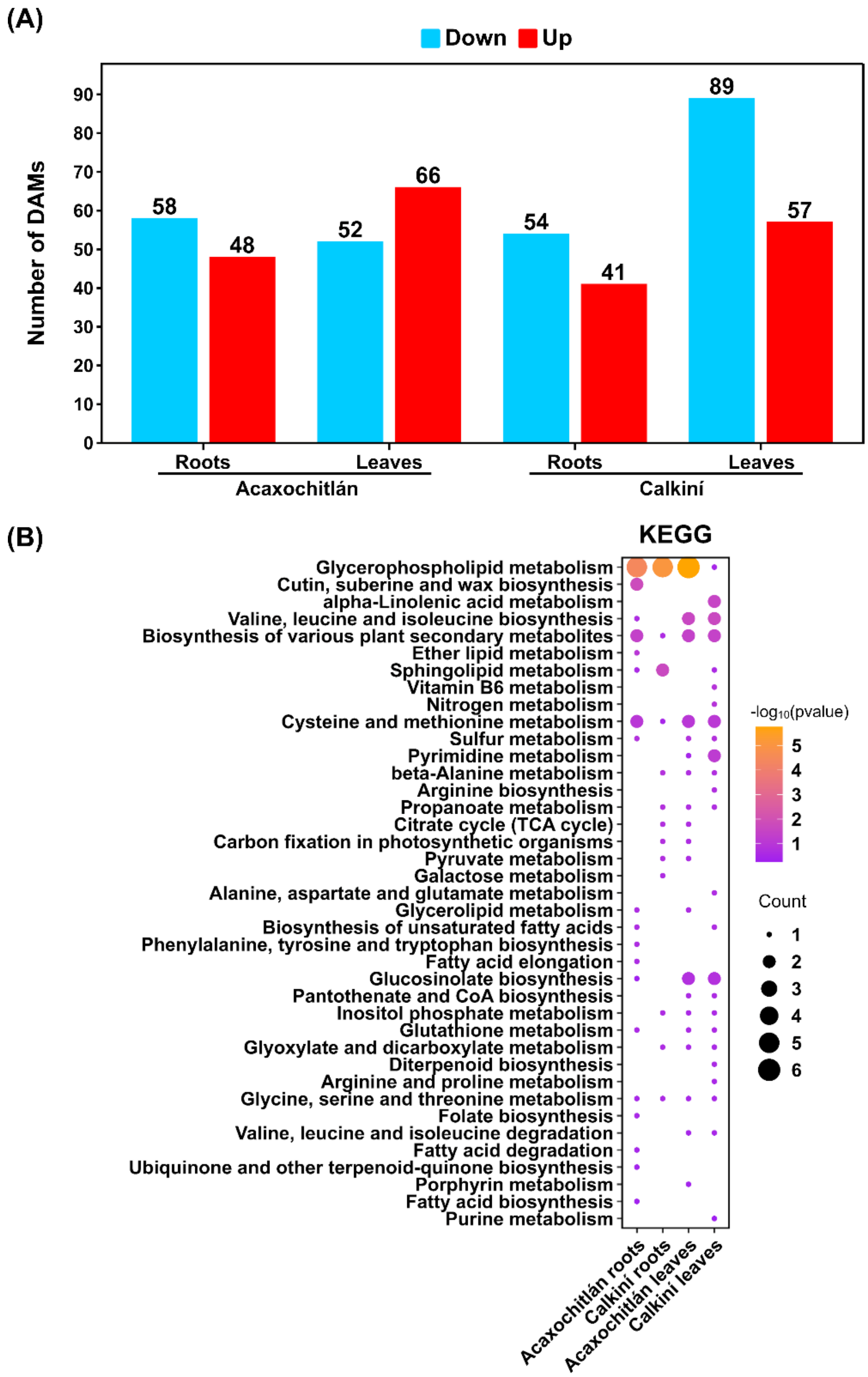

Figure S3 KEGG analysis of differential metabolites. (A) A number of up-and down-regulation of metabolites under heat stress in roots and leaves of both ecotypes. (B) KEGG analysis of tomato up-regulated metabolites under heat stress in roots and leaves of Acaxochitlán and Calkiní ecotype under heat stress based on metabolomics. gProfiler KEGG enriched terms for up-regulated metabolites (log(FC) > 1).

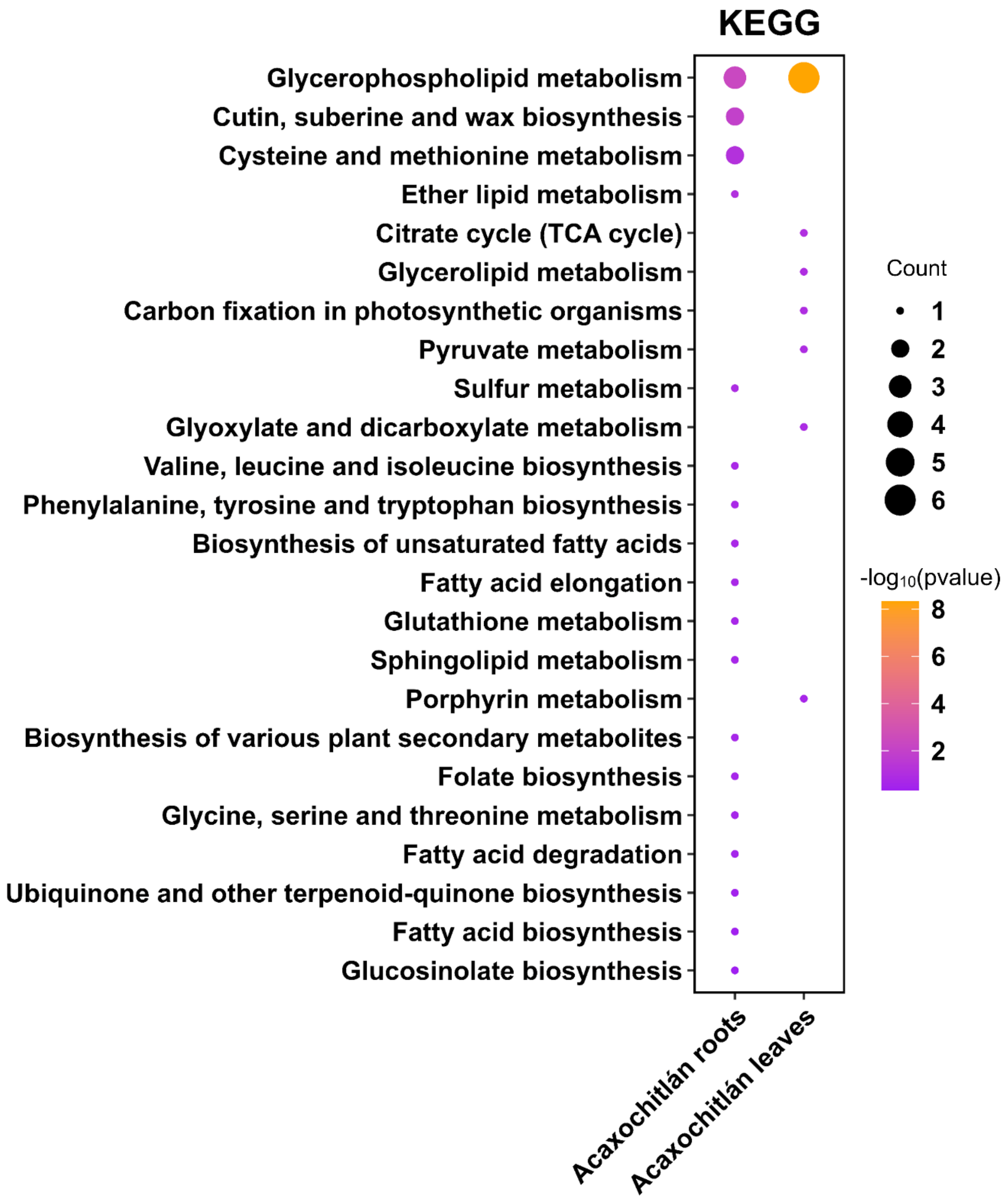

Figure S4 KEGG analysis of tomato up-regulated metabolites under heat stress in roots and leaves of Acaxochitlán ecotype. gProfiler KEGG enriched terms for up-regulated metabolites (log (FC) > 1).
